# Progress in Psychiatry

**Published:** 1903-06-13

**Authors:** 


### PROGRESS IN PSYCHIATRY.

**Toxic Psychoses.**—Dr. J. Galiana, of Madrid, in a paper[1] read before the recent International Medical Congress (section of Neurology and Psychiatry) referred to cases of chronic alcoholism and other psychoses of toxic origin in which mental degeneration pre-exists and co-exists as a complicating factor. In such cases the manifestations of chronic and of acute alcoholism are noticeably different from the pure types. In some of these cases acute attacks may occur which resemble the epileptic excitement and post-epileptic automatism (suspension of mental faculties) of epileptics. The disturbances of chronic alcoholism are also accentuated, and terminal dementia is said to occur early. Instead of the "motor system," it is the "psychical system," as the point of least resistance, which first breaks down in alcoholic degenerates. Alcoholism tends to accentuate and develop all previously existing psychical anomalies. Thus in epileptics it multiplies the number of attacks but is never a cause of the disease *per se*. In hereditarily neurotic or degenerate individuals alcohol has a special and rapid tendency to produce delirium; a single debauch may thus precipitate a typical delirium in such cases. The general constitutional toxæmia of secondary syphilis often produces psychical aberrations in degenerates. Probably three factors co operate in varying degrees to bring about such results, viz., the mental anxiety of the infected patient, the anæmia of syphilis, and the special toxæmia acting upon the neurons or nerve-cells. True syphilitic psychoses are perturbations of the order of delirium, of dementia, and of the motor functions. The motor disturbances may be either paralytic, convulsive, tremulous or ataxic. Motor disturbances of syphilitic origin may occur within the first few years after infection. They usually disappear under anti syphilitic treatment, but some of them may persist.

**Insanity and Delirium from Hasheesh or Cannabis Indica.**—Dr. C. Binet Sangle[2] gives an account of the result of taking a 20 centigramme pill of Cannabis Indica (French pharmacopœia) followed by a cup of coffee. The experiences gone through at the time were written to dictation by a friend. A series of nervous disturbances of an intense degree followed. Movements became jerky, uncertain, almost ataxic, and they increased in number. Sensation was altered, some of the sensations being rendered more acute and fine, while others were diminished. Marked cerebral over-excitement was felt. Dr. Sangle refers to other authors (Dr. Voisin, von Schroff, and the novelist, Theophile Gautier), who have described the effects of self-dosage with Cannabis Indica. Dr. John Warnock, Medical Director of the Egyptian Hospital for the Insane, Cairo, gives a detailed account[3] of insanity resulting from the consumption of Cannabis Indica. Ibn Beitar, an Arabic physician, is said to have been one of the first to recognise an insanity arising from the use of Indian hemp. The physiological symptoms produced by Cannabis Indica included dryness of the mouth, paræsthesia and weakness in the legs, and an inhibition of self-control; the subject wandered about and felt very happy, the time sense became impaired and minutes seemed like hours. The subject laughed and seemed to see the comic side of things, there were lucid intervals which occasionally seemed voluntary, the speech was slurring and the gait ataxic, there was no sleepiness observed. The pulse increased in rate, sensibility was lessened, the face became pale and the pupils were dilated, but there were no hallucinations. In other experiments there was sickness, loss of the time-sense, debility, and increase of appetite. Dr. D. D. Cunningham made experiments on a monkey which was compelled to inhale smoke from Cannabis Indica habitually for eight months with the following results: the animal plainly suffered from hallucinations of sight, and it acquired a positive liking for the drug. Although its appetite decreased it put on fat. The inhalation made the animal drowsy and unsteady in gait, and occasionally convulsions and loss of consciousness resulted. It is noteworthy that hallucinations persisted after other symptoms of intoxication had disappeared. Large quantities of hasheesh, adds Dr. Warnock, are consumed by the inhabitants of Egypt although the importation of the drug is prohibited by law. Most of the drug is smoked in the "gozeh," and in cigarettes, but a considerable amount is eaten in pill form, and in sweetmeats, etc. The usual reason given by patients for taking hasheesh is that it induces a general feeling of pleasure and content. It relieves feelings of lassitude and depression, and also stimulates the sexual appetite when taken in pills or in sweatmeats. The diagnosis of insanity from hasheesh depends upon the history of the case, the discovery of the drug concealed in the patient's clothing or in his ears or mouth, and on the symptoms. Insanity from hasheesh belongs to the toxic group of insanities, like the insanities from alcohol, opium, morphia, and cocaine. It may occur in the following four forms, viz. :—

1. Temporary intoxication, not unlike mild alcoholic intoxication.
2. Delirium from hasheesh, which is accompanied by hallucinations of the senses, and by delusions.
3. Mania from hasheesh, varying in degree of intensity from a mild short attack to a prolonged and furious attack of excitement ending in exhaustion or even death.
4. Chronic mania from hasheesh, a form of chronic mental exaltation, sometimes complicated with delusions of persecution by unseen and malign agencies.

In the temporary intoxication of hasheesh the smoker becomes dull and drowsy, he feels pleasantly exalted, and the worries of life are temporarily blotted out ; fatigue is no longer felt, and he is at peace with the world. The drug acts as a stimulant and a sedative. "This state is to be observed among the *habitués* of hasheesh cafés ; such cases do not come to the asylum." Pleasant half-waking dreams, not unlike those of the opium-taker, occupy the mind, and often the individual feels that he is (temporarily) some important personage. The active excitement of alcoholic inebriety is uncommon, but if the hasheesh smoker is annoyed or interfered with during his dreams he is liable to become irritable and excited, and to show loss of self-control. A staggering gait makes the condition not unlike that of alcoholic intoxication, while the pleasant dreamy state approaches that of the opium smoker. Contrasting the three intoxications, it may be said that the mental pose of the hasheesh smoker is more "subjective" than that of the alcoholic, and less so than that of the absorbed opium user. The alcoholic is the most "objective" and demonstrative of the three. The delirium of hasheesh is accompanied by hallucinations of sight, hearing, taste, and smell, often of an unpleasant kind. Delusions of persecution often occur. The idea that the subject is possessed by a devil or spirit is common. Great exaltation and transformation of the sense of identity may occur. Suicidal impulses are rare. Restlessness and sleeplessness are marked symptoms, but are not so intense as in alcoholic delirium tremens, while the physical exhaustion and the gastro-intestinal disorders of the latter do not occur in hasheesh delirium. The mania of hasheesh is attended with exaltation and notions of personal grandeur, restlessness, incoherence, destructiveness, indecency, and loss of moral feelings and affections. "A certain impudent dare-devil demeanour is a characteristic symptom." Hallucinations of smell and of taste, and delusions of poisoning are not uncommon. There is no pathognomonic symptom of hasheesh mania, but the transitory nature of many cases is often a guide. Cases of hasheesh mania gradually tend towards chronic dementia, with apathy, loss of memory, and the development of dirty and degraded habits. The term *cannabinomania* has been used to describe the mental condition of many hasheesh users between the attacks of their special delirium or mania. Such persons are by nature lazy, shameless, lying, and lead loose lives. While in the asylum they are notorious for making false charges, refusing to work, and quarrelling. The craving for hasheesh in these patients is not so keen as that of a dipsomaniac for alcohol, or a morphinomaniac for opium. No "abstinence phenomena" are noticeable, as in cases of the latter ; and therefore the cessation of the hasheesh habit should be easier than in the case of alcohol or opium, as it actually is. A cannabinomaniac may act as a criminal, and his moral lapses will land him in gaol. Later, when mental impairment becomes obvious, he is usually sent to an asylum. The similarity between cannabinomania and dipsomania is evident ; many of the differences are due to racial peculiarities. Contrasting generally hasheesh insanities with those produced by alcohol, the following conclusions are reached :—First, that suicidal intentions are common among alcoholic and rare among hasheesh cases ; secondly, that hasheesh seems to be a more important factor in the production of insanity in Egypt than alcohol is in England ; thirdly, that as a cause of crime hasheesh appears to be as important in Egypt as is alcohol in England. "It has been suggested," adds Dr. Warnock, "that if the use of hasheesh were entirely prevented in Egypt its place would probably be taken by another stimulant, probably alcohol. Would this change be for the better ? I am inclined to answer in the negative . . . its substitution for hasheesh in Egypt would probably result in a worse state of things." Alcohol also seems to have a specially deleterious effect in warm climates and on Oriental races.

[1] New York Med. News, May 2, 1903. [2] Revue Scientifique, March, 1901. [2] Jour. of Mental Science, Jan., 1903.

